# Mitochondrial function in thoracic aortic aneurysms

**DOI:** 10.1093/cvr/cvy180

**Published:** 2018-07-06

**Authors:** Emma Yu, Kirsty Foote, Martin Bennett

**Affiliations:** Division of Cardiovascular Medicine, University of Cambridge, Addenbrooke’s Hospital, Box 110 ACCI, Cambridge, UK

**This editorial refers to ‘Decreased mitochondrial respiration in aneurysmal aortas of Fibulin-4 mutant mice is linked to PGC1A regulation’ by I. van der Pluijm *et al.*, pp. 1776–1793.**

Mitochondria contain multiple copies of mitochondrial DNA (mtDNA) that encode ribosomal and transfer RNAs and many essential proteins required for oxidative phosphorylation. Mitochondria are essential for generation of adenosine triphosphate (ATP), but also generate reactive oxygen species (ROS) as a by-product of the electron transport chain. Oxidative damage to mtDNA induces respiratory chain dysfunction, resulting in reduced ATP synthesis and further increased ROS generation. The reduced mitochondrial respiration may be accompanied by increased glycolysis and increased lactate production, and these changes can be detected by reduced oxygen consumption and increased extracellular pH of tissues or cultured cells.

Mitochondrial dysfunction leading to reduced mitochondrial respiration has been implicated in both normal vascular ageing and a variety of cardiovascular diseases, including atherosclerosis, heart failure, and aneurysm formation. For example, reduced mtDNA copy number, mitochondrial respiration and expression of specific electron transport chain complexes has been shown in vascular smooth muscle cells (VSMCs) derived from human atherosclerotic plaques.^1–3^ Mitochondriogenesis and mitophagy are also important regulators of mitochondrial health and number. ROS induce VSMC mitophagy, and similarly plaque VSMCs show increased mitophagy compared with normal arterial VSMCs.^2^

Mitochondrial dysfunction has been associated with arterial aneurysm formation, but most studies have analysed abdominal aortic aneurysms (AAAs) rather than genetic thoracic aneurysmal aorta syndromes. For example, AAAs show differential expression of a number of genes associated with mitochondrial function and oxidative phosphorylation^4^ and peroxisome proliferator-activated receptor gamma coactivator 1-alpha (PGC1α) gene expression is decreased in human AAA and angiotensin (Ang) II-induced AAA in mice. Similarly, the intima/media layer of AAA vessels showed reduced expression of a number of markers of mitochondrial biogenesis, including PGC1α, ATP synthase, and citrate synthase.^5^ Mitochondrial dysfunction is also associated with arterial ageing and medial degeneration in humans and mice, associated with changes in arterial expression of genes that regulate mitochondrial number, while correction of mitochondrial dysfunction can delay arterial ageing.^6^ However, mitochondrial dysfunction has not been associated with thoracic aneurysmal aorta syndromes, and its role in causing rather than a consequence of aneurysm formation is unclear.

Fibulin-4 is a secreted glycoprotein which tethers elastic fibres to VSMCs, and patients with mutations in Fibulin-4 develop aortic aneurysms, arterial tortuosity, and elastin abnormalities. Fibulin-4R/R mice (which have four-fold reduced Fibulin 4 levels) develop progressive ascending aneurysm formation and early death around 3 months of age, with Transforming growth factor β (TGFβ) signalling a critical regulator of aneurysm formation.^7^ van der Pluijm *et al*.^8^ used proteomics, genomics, and functional experiments to study thoracic aortas of aneurysmal Fibulin-4R/R animals. Fibulin-4R/R mice showed alterations in protein composition affecting predominantly extracellular matrix (ECM) and cytoskeleton proteins, but also mitochondrial proteins and up-regulation of TGFβ1. VSMCs from Fibulin-4R/R mice had smaller mitochondria, but similar numbers of mitochondria and increased expression of mitochondrial complexes I–IV. VSMCs derived from these mice also showed lower uncoupled oxygen consumption rates, consistent with reduced mitochondrial respiration, but increased acidification rates, perhaps reflecting increased glycolysis. Aortas from aneurysmal Fibulin-4R/R mice also displayed increased ROS levels. Interestingly lower oxygen consumption was also found in Tgfbr-1^M318R/+^ mouse VSMCs, a mouse model for Loeys–Dietz syndrome, and human fibroblasts from Marfan (FBN1) and Loeys-Dietz syndrome (TGFBR2 and SMAD3) patients. Fibulin-4R/R mouse aortas had significantly decreased mRNA expression of Peroxisome PGC1α and PGC1β compared with Fibulin-4+/+ aortas, and activity of PGC1α, a key regulator of both mitochondrial function and organismal metabolism, was markedly reduced. Interestingly, TGFβ also reduced PGC1α transcription, while activation of PGC1α increased both basal and maximum oxygen consumption in Fibulin-4R/R VSMCs and improved cell proliferation.

The authors outline a possible pathway linking Fibulin 4 reduction with mitochondrial dysfunction that includes ROS, TGFβ, and PGC1α (*Figure 1*). Thus, Fibulin deficiency leading to disorganized ECM proteins can result in increased TGFβ1 signalling. ROS may induce mtDNA damage and mitochondrial dysfunction, characterized by reduced mitochondrial respiration, a switch of energy substrate for ATP generation, and further increases in ROS. Mitochondrial biogenesis involves an intricate, complicated network of transcription factors that activate target genes encoding enzymes of fatty acid oxidation, oxidative phosphorylation, and anti-oxidant defences. PGC-1α regulates expression of this network, and directly links external physiological stimuli to the regulation of mitochondrial biogenesis and function. Mitochondrial dysfunction would normally result in compensatory mitogenesis, but increased TGFβ1 signalling due to concomitant abnormalities in either ECM proteins or cytoskeletal filaments result in reduced expression and transcriptional activity of PGC1α, and reduced expression and activation of its targets including PPARα, PPARγ and PPARβ. PPARα and γ. have major roles regulating expression of proteins involved in extra- and intra-mitochondrial fatty acid transport and oxidation, whereas PPARβ regulates antioxidant synthesis, such that reduced expression/activity may increase ROS further.

**Figure 1:**
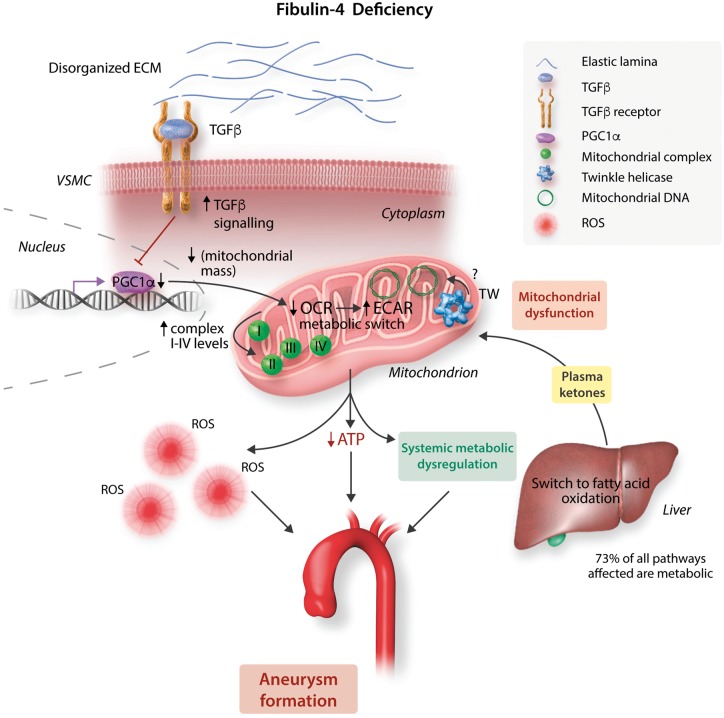
Model implicating mitochondrial dysfunction in aortic aneurysm formation. A full description is provided in the text. ECM, extracellular matrix; VSMC, vascular smooth muscle cell; TGFβ, transforming growth factor beta; PGC1α, peroxisome proliferator-activated receptor gamma coactivator 1 alpha; OCR, oxygen consumption rate; ECAR, extracellular acidification rate; Tw, Twinkle helicase; ROS, reactive oxygen species; ATP, adenosine triphosphate.

As always, the current paper raises a number of important questions. First, how does a deficiency in a protein involved in ECM structural integrity lead to altered mitochondrial function and metabolism. Although this might be through structural abnormalities in mitochondria, TGFβ signalling (which has been implicated in a number of human aneurysm syndromes) negatively regulates PGC1α levels^9^^,^^10^ and may be the major pathway involved. However, arterial ageing, itself associated with ROS and VSMC loss and cell senescence, is accompanied by reductions in mtDNA copy number, mitochondrial respiration, and expression of PGC1α and a number of other genes regulating mtDNA synthesis.^6^ The changes noted in Fibulin-4R/R mice may therefore also be due to loss and/or senescence of VSMCs in the arterial wall, with reduced mitochondrial function and increased ROS. MtDNA synthesis is directly regulated by a number of proteins, including the mitochondrial transcription factor A, mitochondrial helicase Twinkle, and PGC1α. It would therefore be important to examine mtDNA copy number and mtDNA damage in both mouse models of thoracic aortic aneurysm syndromes and human patients, and markers of mtDNA synthesis and mitochondrial turnover including fission/fusion and mitophagy. In addition, we currently lack effective treatments to prevent arterial expansion and rupture in thoracic aortic aneurysm syndromes, and this article raises the possibility that therapy aimed at increasing PGC1α or potent PPARα and γ agonists might be of benefit. If Fibulin-4R/R mice recapitulate the structural abnormalities, mitochondrial dysfunction, and cell signalling defects seen in human patients, then preclinical studies using these agents would be important.

**Conflict of interest:** none declared.

## Funding

This work was supported by British Heart Foundation (BHF) grants [PG/14/69/31032 and RG/13/14/30314], the National Institute for Health Research Cambridge Biomedical Research Centre, the BHF Centre for Research Excellence, and the Academy of Medical Sciences.
